# The utilization of formal and informal home care by older patients with cancer: a Belgian cohort study with two control groups

**DOI:** 10.1186/s12913-017-2594-4

**Published:** 2017-09-12

**Authors:** Abdelbari Baitar, Frank Buntinx, Tine De Burghgraeve, Laura Deckx, Paul Bulens, Hans Wildiers, Marjan van den Akker

**Affiliations:** 1 Department of Oncology, ZNA Middelheim, Antwerp, Belgium; 2 Academic Centre for General Practice/ Department of Public Health and Primary Care, KU Leuven, Leuven, Belgium; 3 Department of Family Medicine, School CAPHRI, Maastricht University, Maastricht, The Netherlands; 4 Department of General Practice, University of Queensland, Brisbane, Australia; 5 Limburgs Oncologisch centrum, Jessa hospital, Hasselt, Belgium; 6 Department of General Medical Oncology, University hospitals Leuven, Leuven, Belgium

**Keywords:** Formal care, Informal care, Home care, Cancer, Older patients

## Abstract

**Background:**

The purpose of this paper is to analyse the utilization of formal and informal home care among older patients with cancer (OCP) and to compare this with middle-aged patients with cancer (MCP) and older patients without cancer (ONC). Additionally, we examined predictors of transitions towards formal care one year after a cancer diagnosis.

**Methods:**

OCP and MCP had to be recruited within three months after a cancer diagnosis and have an estimated life expectancy over six months. ONC consisted of patients without known cancer, seen by the general practitioner. Formal and informal care were compared between the patient groups at baseline, i.e. shortly after a cancer diagnosis and changes in care were studied after one year.

**Results:**

A total of 844 patients were evaluable for formal care at baseline and 469 patients (56%) at follow-up. At baseline, about half of older adults and 18% of MCP used formal care, while about 85% of cancer patients and 57% ONC used informal care. Formal care increased for all groups after one year though not significantly in OCP. The amount of informal care only changed in MCP which decreased after one year. Cancer-related factors and changes in need factors predict a transition towards formal care after a cancer diagnosis.

**Conclusions:**

A cancer diagnosis has a different impact on the use of formal and informal care than ageing as such. The first year after a cancer diagnosis is an important time to follow-up on the patients’ needs for home care.

**Electronic supplementary material:**

The online version of this article (10.1186/s12913-017-2594-4) contains supplementary material, which is available to authorized users.

## Background

The increasing population of older people and changes in health policy have resulted in a shift from institutional care to home care. It is obvious that functional status plays an important role in the use of professional home care. Therefore, in Belgium and other countries planning and financing of both home nursing and nursing homes are largely based on levels of functioning [1]. Other factors known to be associated with professional home care use include age, gender, educational level, marital status, and informal care [2–5]. Most of this evidence was based on cross-sectional analyses comparing older people already using professional home care with non-users. Kempen et al. specifically compared older people who started to use professional home care to matched non-users which is more appropriate to understand factors that explain the use of professional home care [2]. Geerlings et al. focused on the process of becoming a user of informal and professional home care by studying transitions in the use of care based on longitudinal data in the Netherlands [6]. More recent studies, based on the Survey of Health, Ageing and Retirement in Europe (SHARE) data, showed cross-national differences in the dynamics of care between various European countries [7–9]. In the present study, we focused on a specific population, Flemish older patients with cancer (OCP). The rising population of older people is accompanied by an increase of cancer prevalence rates. A diagnosis of cancer and subsequent treatment can have a substantial effect on the well-being of patients and their need for home care. As shown in our recent work, patients with cancer have increased levels of depression, loneliness, and increasing difficulties in cognitive functioning over the course of one year [10, 11]. In light of this, we expect to see significant transitions in care after a diagnosis of cancer. The purpose of this paper is to analyse the utilization of formal and informal home care among OCP shortly after diagnosis and to examine changes after one year. The OCP will be compared to two control groups, a group of middle-aged patients with cancer (MCP) and a group of older primary care patients without cancer (ONC). A second goal is to examine predictors of transitions from no formal care to formal care following a cancer diagnosis.

## Methods

### Study design and population

This analysis was performed on baseline and one-year follow-up data from the Klimop study, which is an ongoing study in Belgium and the Netherlands on the impact of cancer, aging, and their interaction on the well-being of OCP. For this purpose, OCP are compared with MCP (aging effect) and with ONC (diagnosis effect). The same design was used for the current analysis on home care, however based on our results it sometimes made more sense to compare all patients with cancer to ONC in the discussion section. Full details of the Klimop study have been described elsewhere [12]. In short, OCP (≥ 70 years), MCP (50-69 years), and ONC (≥ 70 years) are longitudinally compared for different measures of well-being. The group of cancer patients consisted of patients with breast, gastro-intestinal, and lung cancer. Patients had to be recruited within three months after a cancer diagnosis and had to have an estimated life expectancy of more than six months. Data have been collected through personal interviews at baseline (T0), after one year (T1), and subsequently every two years. The analysis for this paper was restricted to patients living at home and who were recruited in Belgium only, given the different homecare system in the Netherlands.

### Measurements

#### Formal care

Professional home care or formal care was dichotomized in ‘users’ and ‘non-users’. Users were defined in this study as having received help from at least one of the following paid professionals in the last three months: home nurse, home help services, physiotherapist, meals on wheels, adult day care, and cleaning help. Formal care was only recorded if the participants had at least five contacts in the last three months prior to the interview in order to avoid the measurement of sporadic use and to be able to evaluate recent use.

#### Informal care

Participants were asked to indicate who cared for them, apart from professional help. This could be a partner, children, other relatives, friends, neighbours, or volunteers. Informal care was defined as help provided by any of them (partners, children, other relatives, friends, neighbours, or volunteers).

#### Independent variables

Consistent with previous research on home care, we used Andersen and Newman’s behavioural model as a theoretical framework to order variables at the individual level in predisposing, enabling and need factors to predict the utilization of formal care [13]. Predisposing variables were: age, gender, marital status, and educational level. We considered the availability of informal care as an enabling variable. As need factors we considered functional status, depression, loneliness, fatigue, cognitive status, nutrition, polypharmacy and comorbidity. Since the focus of this study is on cancer, we also evaluated the cancer-related factors tumour type, stage, and treatment.

Functional status was measured with the Katz index of Activities of Daily Living (ADL) (range: 0-6) and the Lawton Instrumental ADL (IADL) scale (range: 0-8 for woman, range: 0-5 for men) [14, 15]. Dependence in one or more domains for each test was defined as having an impaired test result. Depression was measured using the Geriatric Depression Scale (range: 0-15, cut-off ≥5), loneliness with the loneliness scale of De Jong-Gierveld (range: 0-11, cut-off ≥3), fatigue with a Visual Analogue Scale (range: 0-10, cut-off ≥4), cognitive status with the Mini Mental State Examination (range: 0-30, cut-off <24), and comorbidity with the Charlson Comorbidity Index (CCI) (range: 0-37, cut-off ≥1) [16–19]. Nutrition was measured with a new and adapted version of the Mini Nutritional Assessment-short form (range: 0-14, cut-off ≤11), which is currently being validated by our group [20].

### Data analysis

Firstly, formal and informal care were studied separately. Individual home care services, as specified in our definition of formal care, were studied as well. Finally, we considered formal care and informal care simultaneously. This entails four possible situations at baseline: no care, informal care only, formal care only, and the availability of both formal and informal care. Hence, 16 alternative transitions are possible after one year. Next to transitions to formal care or informal care, we also evaluated whether both types of care substitute (‘substitution’) or complement (‘complementarity’) each other to better understand the relationship between formal and informal care. Substitution was defined as the sum of transitions from formal to informal care and vice versa. Complementarity was defined as the sum of transitions from no care or (in)formal care only towards combined formal and informal care.

We performed comparative analyses between OCP and two control groups ONC and MCP at baseline by means of the chi-square test. Changes in care over time were studied by comparing formal and informal care between baseline and one year follow-up within each patient group with the McNemar test and by calculating the percentages of every transition in and between formal and informal care. We set alpha at 0.05 for all analyses to denote statistical significance.

For the second goal of our analysis, logistic regression analyses were conducted to explain the transition of no formal care to formal care in patients with cancer, both OCP and MCP. Separate analyses for OCP and MCP were not conducted due to a small sample size for OCP. Univariate analyses were performed with all predisposing, enabling, and need factors as continuous predictors with the exception of IADL which was analyzed as a dichotomous variable due to the different score range for women and men. In addition, we considered changes of need factors over time by dichotomizing them and identifying changes in categories between baseline and one year follow-up. All analyses were performed using SPSS 23 software (Chicago, IL).

## Results

### Study population

**Fig. 1:**
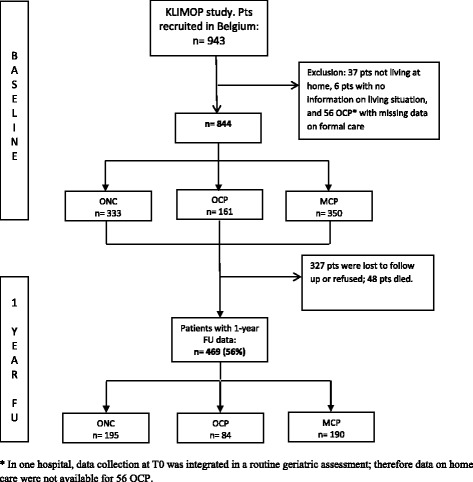
The utilization of formal care: Patient flow chart

A total of 844 patients, recruited between April 2010 and November 2013, were available for analysis at baseline. At follow-up, data for formal care were also available for a total of 469 patients (56%) (see Fig. 1). Missing follow-up data were due to death (2.7% ONC, 10.6% OCP, and 6.3% MCP) or to loss of follow-up/refusal (38.7%, 37.3%, and 39.4%). Patient characteristics of OCP and the two control groups are shown in Table 1. Differences in need factors for formal care are found in Table 2. The majority of the patients were female (ONC: 61.3%, OCP: 69.6%, MCP: 75.1%). Compared to ONC, OCP had a worse nutritional status, were less lonely, and had less comorbidity and polypharmacy.

**Table 1:** Patient characteristics of the three patient groups at baseline

	ONC			OCP			MCP	
	n	%	*p*-value	n	%	*p-value*	n	%
Total N° pts	333	100.0		161	100.0		350	100.0
Age								
Mean (SD)	78.7 (5.7)			76.9 (5.0)			59.8 (5.4)	
Gender			0.07			0.19		
Female	204	61.3		112	69.6		263	75.1
Male	129	38.7		49	30.4		87	24.9
Living situation			0.75			*< 0.001*		
Alone	109	32.7		55	34.2		48	13.7
Not alone	224	67.3		106	65.8		302	86.3
Marital status			0.68			*< 0.001*		
Married/living together	209	62.8		98	60.9		285	81.4
Unmarried/widow/divorced	124	37.2		63	39.1		65	18.6
Educational level	*n* = 328		0.66	*n* = 157		*< 0.001*	*n* = 344	
≤ 14 years	94	28.7		50	31.8		37	10.8
15-19 years	150	45.7		72	45.9		161	46.8
≥ 19 years	84	25.6		35	22.3		146	42.4
Tumour type						0.07		
Breast				86	53.4		217	62.0
Gastrointestinal				69	42.9		114	32.6
Lung				6	3.7		19	5.4
Stage				*n* = 137		*0.004*	*n* = 327	
I				18	13.1		87	26.6
II				69	50.4		121	37.0
III				35	25.5		93	28.4
IV				15	10.9		26	8.0
Treatment^a^								
Surgery^b^				132	89.8	0.77	304	88.9
Chemotherapy^b^				63	43.4	*< 0,001*	210	61.6
Radiotherapy^b^				71	49.3	*< 0,001*	232	68.0
Hormonal^b^				59	40.7	*0.03*	175	51.6

OCP were compared to the two control groups with the chi-square test. *p* < 0.05 denotes statistical significance

^a^More than one possibility

^b^Percentages were calculated on valid cases

**Table 2:** Need factors for formal care of the three patient groups at baseline

	ONC			OCP			MCP	
	n	%	*p-value*	n	%	*p-value*	n	%
Total N° pts	333	100.0		161	100.0		350	100.0
ADL			0.80			*< 0,001*		
Independent	184	55.3		87	54.0		273	78.0
Dependent	149	44.7		74	46.0		77	22.0
IADL	*n* = 329		0.55	*n* = 160		*< 0,001*	n = 344	
Independent	184	55.9		94	58.8		269	78.2
Dependent	145	44.1		66	41.3		75	21.8
Depression	*n* = 322		0.53	*n* = 148		0.34	n = 322	
Normal	276	85.7		130	87.8		292	90.7
Impaired	46	14.3		18	12.2		30	9.3
Cognition			0.72			*0.01*		
Normal	292	87.7		143	88.8		333	95.1
Impaired	41	12.3		18	11.2		17	4.9
Nutrition	*n* = 280		*< 0,001*	*n* = 135		0.45	*n* = 320	
Normal	229	81.8		48	35.6		102	31.9
At risk/malnourished	51	18.2		87	64.4		218	68.1
Loneliness	*n* = 318		*0.004*	*n* = 138		*0.03*	n = 322	
Not lonely	178	56.0		97	70.3		257	79.8
Lonely	140	44.0		41	29.7		65	20.2
Fatigue	*n* = 331		0.19	*n* = 149		0.18	*n* = 336	
No fatigue	150	45.3		58	38.9		153	45.5
Fatigue	181	54.7		91	61.1		183	54.5
Polypharmacy			*< 0,001*			*< 0,001*		
< 5 drugs	150	45.0		109	67.7		305	87.1
≥ 5 drugs	183	55.0		52	32.3		45	12.9
Comorbidity	*n* = 326		*< 0,001*	n = 148		*< 0.001*	*n* = 339	
CCI 0	145	44.5		92	62.2		269	79.4
CCI ≥ 1	181	55.5		56	37.8		70	20.6

OCP were compared to the two control groups with the chi-square test. *p* < 0.05 denotes statistical significance

### The comparison of formal and informal care at baseline

Results of the baseline comparisons between OCP and the two control groups are summarized in Table 3. About half of the OCP were users of formal care (51.6%). There was no difference with the group ONC (50.2%), but significantly less MCP (18.0%) were users.

**Table 3:** The comparison of formal and informal care at baseline between OCP and two control groups

	ONC			OCP			MCP	
	*n*	%	*p*-value	*n*	%	*p*-value	*n*	%
Formal care	*n* = 333		0.77	*n* = 161		*< 0.001*	*n* = 350	
Users	167	50.2		83	51.6		63	18.0
Non-users	166	49.8		78	48.4		287	82.0
Individual home care service								
Home nursing	66	19.8	0.86	33	20.5	*< 0.001*	14	4.0
Home help services	24	7.2	0.50	9	5.6	*0.01*	5	1.4
Cleaning help	140	42.0	0.41	74	46.0	*< 0.001*	49	14.0
Physiotherapist	37	11.1	*0.003*	5	3.1	0.62	14	4.0
Meals on wheels	26	7.8	0.24	8	5.0	*< 0.001*	0	0.0
Informal care	n = 331		*< 0.001*	n = 157		0.86	*n* = 335	
Present	188	56.8		135	86.0		286	85.4
Not present	143	43.2		22	14.0		49	14.6
Formal care + informal care	*n* = 331			n = 157			n = 335	
No care	84	25.4	*< 0,001*	11	7.0	0.13	38	11.3
Informal care only	80	24.2	*< 0,001*	66	42.0	*< 0,001*	237	70.7
Formal care only	59	17.8	*0.001*	11	7.0	0.06	11	3.3
Informal care + formal care	108	32.6	*0.02*	69	43.9	*< 0,001*	49	14.6

OCP were compared to the two control groups with the chi-square test. *p* < 0.05 denotes statistical significance

The analysis of the individual home care services only show a significant difference (*p* = 0.003) for seeking help from a physiotherapist between OCP (3.1%) and ONC (11.1%). Compared to MCP, we observed no difference for help from the physiotherapist but OCP made significantly more use for all other home care services. None of the patients made use of adult day care. An overview of the number of home care services per patient at T0 is shown in Additional file 1. While most MCP received 1 or 2 individual home care services, more than 10% of the OCP and ONC relied on 3 or more services.

Furthermore, our results showed that OCP (86.0%) could rely as much on informal care as MCP (85.4%) and more so than ONC (56.8%).

When considering both formal and informal care, the distribution of types of care differed in every aspect between OCP and ONC. ONC had a higher proportion (25.4%) of patients with no care compared to the two cancer cohorts (OCP: 7.0%, MCP: 11.3%). MCP had more informal care only (70.7%) than OCP (42.0%) while OCP relied more on both types of care (43.9%) than MCP (14.6%).

### The comparison of formal and informal care between T0 and T1

Table 4 presents the differences in care between T0 and T1 in the three patients groups. In the group ONC, there was overall an increase of formal care at T1. No other differences were observed.

**Table 4:** The comparison of formal and informal care between T0 and T1 in each patient group

	ONC					OCP					MCP				
	T0		T1			T0		T1			T0		T1		
	*n*	%	*n*	%	*p*-value	n	%	*n*	%	*p*-value	*n*	%	*n*	%	*p*-value
Formal care	*n* = 333		*n* = 195		*0.03*	*n* = 161		*n* = 84		0.06	*n* = 350		*n* = 190		*< 0,001*
Users	167	50.2	110	56.4		83	51.6	55	65.5		63	18.0	77	40.5	
Non-users	166	49.8	85	43.6		78	48.4	29	34.5		287	82.0	113	59.5	
Individual home care service															
Home nursing	66	19.8	42	21.5	0.08	33	20.5	20	23.8	1.00	14	4.0	21	11.1	*0.01*
Home help services	24	7.2	9	4.6	0.21	9	5.6	6	7.1	0.73	5	1.4	3	1.6	1.00
Cleaning help	140	42.0	92	47.2	0.29	74	46.0	43	51.2	1.00	49	14.0	44	23.2	*0.02*
Physiotherapy	37	11.1	23	11.8	0.66	5	3.1	16	19.0	*0.01*	14	4.0	34	17.9	*< 0,001*
Meals on wheels	26	7.8	11	5.6	0.11	8	5.0	3	3.6	1.00	0	0.0	1	0.5	1.00
Informal care	n = 331		*n* = 191		1.00	n = 157		*n* = 80		0.65	n = 335		*n* = 185		*0.03*
Present	188	56.8	103	53.9		135	86.0	63	78.8		286	85.4	140	75.7	
Not present	143	43.2	88	46.1		22	14.0	17	21.3		49	14.6	45	24.3	
Formal care + informal care	n = 331		n = 191			n = 157		n = 80			n = 335		n = 185		
No care	84	25.4	40	20.9	0.10	11	7.0	7	8.8	1.00	38	11.3	30	16.2	0.24
Informal care only	80	24.2	43	22.5	1.00	66	42.0	19	23.8	*0.01*	237	70.7	79	42.7	*< 0,001*
Formal care only	59	17.8	48	25.1	0.07	11	7.0	10	12.5	0.79	11	3.3	15	8.1	0.08
Informal care + formal care	108	32.6	60	31.4	1.00	69	43.9	44	55.0	0.06	49	14.6	61	33.0	*< 0,001*

Proportions between T0 and T1 were compared with the McNemar test. *p* < 0.05 denotes statistical significance

In the group OCP, there was overall an increase of formal care at T1. However this difference was not statistically significant (*p* = 0.06). At the level of individual home care services, significantly more patients saw a physiotherapist after one year (from 3.1% to 19.0%, *p* = 0.01). There was no significant difference in informal care between T0 and T1 (*p* = 0.65). However, when considering both formal and informal care, fewer patients received only informal care (p = 0.01) at T1.

In the group MCP, there was overall an increase of formal care at T1. Increased help from a home nurse (from 4.0% to 11.1%), cleaning help (from 14.0% to 23.2%), and a physiotherapist (from 4.0% to 17.9%) was reported. From the patients who received physiotherapy, 71.4% and 94.1% had breast cancer at respectively T0 and T1. Informal care decreased at T1 (from 85.4% to 75.7%). When considering both formal and informal care, fewer patients received only informal care and more patients received both formal and informal care.

### Transitions in formal and informal care after one year

Transitions in formal and informal care were analyzed separately in Table 5. There was a significant difference in the transition from no formal care to formal care between OCP and the two control groups. For the group OCP, 16.7% made this transition compared to 8.2% ONC (*p* = 0.04) and 26.8% MCP (*p* = 0.07). For the transition from formal care to no formal care, a similar proportion OCP (6.0%) stopped formal care compared to MCP (6.3%) after one year. Only a small proportion of ONC (2.6%, *p* = 0.16) stopped relying upon formal care.

**Table 5:** Transitions in formal and informal home care analyzed separately

	ONC			OCP			MCP	
	*n*	%	*p*-value	*n*	%	*p*-value	*n*	%
	Formal care							
Transition	*n* = 195			*n* = 84			*n* = 190	
No formal care at T0 and T1	80	41.0	*0.05*	24	28.6	*< 0,001*	101	53.2
Formal care at T0 and T1	94	48.2	0.93	41	48.8	*< 0,001*	26	13.7
No formal care -- > Formal care	16	8.2	*0.04*	14	16.7	0.07	51	26.8
Formal care -- > No formal care	5	2.6	0.16	5	6.0	0.91	12	6.3
	Informal care							
Transition	*n* = 190			*n* = 79			*n* = 178	
No informal care at T0 and T1	62	32.6	*< 0,001*	6	7.6	0.55	10	5.6
Informal care at T0 and T1	79	41.6	*< 0,001*	54	68.4	0.88	120	67.4
No informal care -- > Informal care	24	12.6	0.56	8	10.1	0.77	16	9.0
Informal care -- > No informal care	25	13.2	0.87	11	13.9	0.42	32	18.0

OCP were compared to the two control groups with the chi-square test. *p* < 0.05 denotes statistical significance

Transitions in informal care were similar between OCP and the two control groups. In the groups ONC, OCP, and MCP respectively 12.6%, 10.1%, and 9.0% made a transition to informal care at T1 while respectively 13.2%, 13.9%, and 18.0 stopped relying on informal care.

Transitions considering the availability of formal care and informal care simultaneously are summarized in Additional file 2. Transitions in care, from any type, were observed in ONC, OCP, and MCP in respectively 33.2%, 39.2%, and 49.4% of the patients. The analysis of substitution and complementarity of care shows that in 5.1% of OCP formal and informal care substitute each other, while in 17.7% both types of care complement each other at T1. Results for the control groups are shown in Table 6.

**Table 6:** Substitution and complementarity of formal and informal care

	ONC			OCP			MCP	
	*n*	%	*p*-value	*n*	%	*p*-value	*n*	%
Transition	n = 190			n = 79			n = 178	
Substitution	0	.0	0.07	4	5.1	1.0	11	6.2
Complementarity	19	10.0	0.08	14	17.7	0.44	39	21.9

OCP were compared to the two control groups with the chi-square test or fisher’s exact test where appropriate. *p* < 0.05 denotes statistical significance

### Predictors of the transition no formal care to formal care after a cancer diagnosis

The studied sample for this analysis consisted of a total of 190 patients with cancer (20.0% OCP), of which 65 made the transition from no formal care to formal care. Significant predictors in univariate analysis are shown in Table 7. Next to a higher value for the need factors fatigue and polypharmacy, certain changes (or the lack of) in ADL, IADL, depression, fatigue, and polypharmacy were predictive for a transition towards formal home care. Furthermore, a worse cancer stage and having received chemotherapy or radiotherapy were predictive as well. Factors that were not predictive included predisposing variables, ADL, IADL, informal care, and belonging to the group OCP or MCP.

**Table 7:** Predictors of the transition no formal to formal care 1 year after a cancer diagnosis (OCP and MCP combined, *n* = 190)

	Univariate^a^		
	OR	95% CI	*p*-value
Need factors			
Fatigue	1.24	1,09-1,41	0.001
Polypharmacy	1.18	1,01-1,38	0.04
Cancer-related factors			
Stage			0.09
I	ref		
II	2.01	0,85-4,78	0.11
III	2.67	1,02-6,96	0.05
IV	5.93	1,18-29,68	0.03
Chemotherapy	2.21	1,17-4,19	0.02
Radiotherapy	2.38	1,19-4,77	0.02
Changes in need factors			
ADL			0.01
persistently independent	ref		
became indepedent	0.93	0,27-3,13	0.90
became dependent	3.19	1,55-6,54	0.002
persistently dependent	0.85	0,28-2,54	0.77
IADL			0.04
persistently independent	ref		
became indepedent	1.81	0,60-5,50	0.29
became dependent	2.86	1,36-6,04	0.01
persistently dependent	2.04	0,66-6,33	0.22
Depression			0.06
persistently normal	ref		
became normal	2.53	0,77-8,32	0.13
became depressed	3.03	1,21-7,60	0.02
persistently depressed	2.53	0,34-18,59	0.36
Fatigue			0.003
persistently normal	ref		
became normal	6.00	1,55-23,19	0.01
became impaired	4.00	1,19-13,42	0.03
persistently impaired	8.27	2,61-26,22	< 0,001
Polypharmacy			0.04
persistently normal	ref		
became normal	2.51	0,60-10,52	0.21
became impaired	2.72	1,15-6,46	0.02
persistently impaired	2.93	0,93-9,25	0.07

^a^Predictors of univariate logistic regression analyses are shown with *p* < 0,05

## Discussion

Transitions in home care depend on the situation. A diagnosis of cancer might be considered a situation of greater need for care and as such we observed transitions in formal and informal care in ONC, OCP, and MCP in respectively 33.2%, 39.2%, and 49.4% after one year. Our results also showed an expected increase in new users of formal care in cancer patients: 16.7% for OCP, 26.8% for MCP compared to 8.2% for ONC. However, at baseline, i.e. shortly after diagnosis, some important differences were already observed between the patient groups in terms of care but also in patient characteristics. While many differences between OCP and MCP can be explained due to age-related factors, some differences between OCP and ONC were less obvious. OCP had a lower comorbidity burden and polypharmacy. This can partially be explained by a referral bias for OCP; the frailest patients are not always referred to the oncologist. OCP reported to be less lonely despite no difference in marital status or living situation. Previously, we already showed that at baseline fewer patients with cancer had feelings of loneliness than ONC and that after one year the proportion of cancer patients with loneliness increased significantly reaching the levels of ONC [11]. Our current analysis might explain these differences in loneliness by looking at the received informal care. More patients with cancer relied on informal care compared to ONC at baseline. This care was likely provided only recently around the time the patient was informed about a cancer diagnosis which could explain the difference in baseline loneliness between patients with cancer and ONC. Furthermore, informal care decreased after one year in both cancer cohorts, although not significantly in OCP, which could contribute to the increase in loneliness in both cancer cohorts. It is to be expected that newly diagnosed patients receive much help and support from their environment at first but less so after completing their treatment and this might have an impact on feelings of loneliness, an important measure of well-being.

The analysis of the individual home care services showed that cleaning help was clearly the most used service in the three patient groups. In this regard, we note that we did not document whether the use of this service was related to any health-related issues. Furthermore, at baseline fewer cancer patients visited the physiotherapist compared to ONC. However, after one year a strong increase in physiotherapy was observed in both cancer cohorts, mainly in patients with breast cancer. This might be related to lymphoedema following breast cancer surgery or radiotherapy. While, besides physiotherapy, no other changes were observed after one year in OCP, MCP made more use of home nursing and cleaning help next to an increase in physiotherapy. A possible explanation for this is that due to lymphoedema, MCP are less able to do household tasks like cleaning or are advised to limit the strenuous use of their arm. Also, an important proportion of patients with cancer will have had an intestinal stoma. These patients, OCP likely more than MCP, might rely upon home nursing for stoma care. These increases of home nursing and cleaning help after one year in MCP are however not observed in OCP. However, the baseline percentages for both home care services are already high in OCP and new tasks might have been covered by the already available care in this group.

About half of the OCP and ONC received formal care at baseline. This formal care was more often in combination with informal care in OCP than in ONC. When considering both formal care and informal care after one year, the main trend observed in both cancer cohorts, in contrast to ONC, is the decrease in the number of patients who rely on informal care only and the increase in patients with formal care whether or not with informal care. This distinction between ONC and cancer patients can be explained by the dynamics in informal care in cancer patients as discussed previously and by the expected increased need for formal care in cancer patients which was quantified in this study. Another important observation is the lack of any change in the proportion of patients with no care whatsoever in the three patient groups.

Several studies in the general population suggest that formal and informal care complement rather than substitute each other [6–8]. This is also shown in our three patient groups. Our rates for substitution and complementarity for ONC are similar with other reports for the general population in Belgium [8]. These rates are however higher in our studied cancer cohorts, particularly for complementarity. In the context of cancer, more technical skills (e.g. injections, stoma care, and physiotherapy) for care might be required and this care will complement rather than substitute informal care. Several care models have been proposed for the general population [21]. For patients diagnosed with cancer, formal and informal care might be better explained with a complementarity and task-specific model.

Our results show different dynamics in care between older patients with and without cancer but also between OCP and MCP. International guidelines recommend the implementation of a geriatric assessment in OCP to guide treatment decisions in routine oncology practice [22]. This assessment will lead to the necessary referrals for many patients with cancer [23]. In contrast to the general population, many OCP will be seen by a social worker in the hospital which will also drive changes in formal care. In this regard, we mention the Belgian implementation of the InterRAI instruments (BelRAI) which is an ambitious web-based comprehensive assessment system to improve the quality and continuity of care across different health care settings, including home care [24–26]. It is advisable to harmonize recommendations in geriatric oncology to implement geriatric assessment in daily practice with efforts like the BelRAI for the general population at a national level for future policies.

For the second goal of our study, we analyzed predictors for the transition from no formal care to formal care one year after a cancer diagnosis. Functional status, the availability of informal care, and dispositional factors did not predict the transition towards formal care in patients with cancer. Cancer-related factors, i.e. more advanced disease or a more extensive treatment, and the related worsening of need factors (more than their baseline values) were significant predictors. In contrast, an analogous analysis on ONC showed that only developing a nutritional impairment predicted a transition towards formal care (data not reported). Our results show that the first year after a cancer diagnosis is an important time to follow-up on the patients’ needs for home care at different time points during the disease trajectory. More longitudinal research is needed to determine to what degree a cancer diagnosis is a turning point towards more formal care or whether the increased use of formal care is more of a temporary nature. Another evolution to follow in the future relevant to homecare, is the parenteral administration of cancer treatments at home. The first pilot projects in this regard have been started in Belgium.

When interpreting our results, some considerations should be made. The study of both formal and informal care is a strength of this study, however the collection of more detailed information on informal care would have been beneficial for our analysis. Many patients were lost to follow-up (LTFU), i.e. a total of 38.7% when not considering the patients that died (5.7%). Additional analyses (Additional file 3) show no major baseline differences between LTFU patients who were alive and patients with one-year follow-up data. LTFU patients had less cleaning help and more informal care only, no other differences were observed for home care. Furthermore, there was a similar proportion of LTFU patients (excluding deaths) in the three patient groups. Another point to consider is that our results apply for Belgium, a well-developed welfare state with well-developed formal services. A lot has been written in the home care literature, which focuses on the general population, about the different dynamics of formal and informal care between European countries, about the influence of the strength of family ties, and a north-south gradient [8]. To our knowledge, there are no similar studies to which we can compare our results with that evaluate changes in both formal and informal care after a cancer diagnosis.

## Conclusions

A cancer diagnosis has a different impact on the use of formal and informal care than ageing as such. The first year after a cancer diagnosis is an important time to follow-up on the patients’ needs for home care.

## Additional files

Additional file 1: Amount of home care services per patient at baseline. An overview of the number of home care services per patient at T0 is shown for the three patient groups. (XLSX 16 kb)

Additional file 2: Individual transitions between formal care and informal care. Transitions considering the availability of both formal care and informal care are summarized for the three patient groups. (XLSX 13 kb)

Additional file 3: Baseline comparisons for care in patients with and without missing 1-year data. Formal care and informal care are compared between patients included in the longitudinal analyses and patients that were lost to follow-up after 1 year. Separate comparisons were made with patients who were lost to follow-up and alive and with patients who had died after 1 year. (XLSX 14 kb)

Additional file 4: Database. The database on which the current analysis was conducted is included. (SAV 57 kb)

## Abbreviations

ADL: Activities of daily living
CCI: Charlson comorbidity index
IADL: Instrumental activities of daily living
LTFU: Lost to follow-up
MCP: Middle-aged patients with cancer
OCP: Older patients with cancer
ONC: Older primary care patients without cancer
